# Minimum volume standards in German hospitals: do they get along with procedure centralization? A retrospective longitudinal data analysis

**DOI:** 10.1186/s12913-015-0944-7

**Published:** 2015-07-22

**Authors:** Werner de Cruppé, Marc Malik, Max Geraedts

**Affiliations:** Institute for Health Systems Research, School of Medicine, Faculty of Health, Witten/Herdecke University, Alfred-Herrhausen-Strasse 50, 58448 Witten, Germany

**Keywords:** Volume-outcome, Minimum volume standards, Centralization, Hospital, Germany, Pancreatectomy, Esophagectomy, Transplantation, Arthroplasty, Replacement, Knee

## Abstract

**Background:**

Compliance with minimum volume standards for specific procedures serves as a criterion for high-quality patient care. International experiences report a centralization of the respective procedures. In Germany, minimum volume standards for hospitals were introduced in 2004 for 5 procedures (complex esophageal and pancreatic interventions; liver, kidney and stem cell transplantations), in 2006 total knee replacement was added. This study explores whether any centralization is discernible for these procedures in Germany.

**Methods:**

A retrospective longitudinal analysis of secondary data serves to determine a possible centralization of procedures from the system perspective. Centralization means that over time, fewer hospitals perform the respective procedure, the case volume in high-volume hospitals increases together with their percentage of the annual total case volume, and the case volume in low-volume hospitals decreases together with their percentage of the annual total case volume. Using data from the mandatory hospital quality reports for the years 2006, 2008 and 2010 we performed Kruskal Wallis and chi-square tests to evaluate potential centralization effects.

**Results:**

No centralization was found for any of the six types of interventions over the period from 2006 to 2010. The annual case volume and the number of hospitals performing interventions rose at differing rates over the 5-year period depending on the type of intervention. Seven percent of esophagectomies and 14 % of pancreatectomies are still performed in hospitals with less than 10 interventions per year.

**Conclusions:**

For the purpose of further centralization of interventions it will be necessary to first analyze and then appropriately address the reasons for non-compliance from the hospital and patient perspective.

## Background

Minimum volumes are still deemed to be a criterion for better quality in patient care for numerous surgical interventions, since many studies and systematic reviews document better outcomes in high-volume hospitals compared to low-volume hospitals [1–16]. A shift of cases from low-volume hospitals to high-volume hospitals is a conclusion of most studies, and in one review is even quantified as number needed to treat [17]. The volume outcome relationship and the centralization of these procedures are therefore two sides of the same coin. In the United States, recommendations of the National Cancer Policy Board of the Institute of Medicine [18] or initiatives such as Leapfrog on Evidence-based Hospital Referral [19] promote centralization towards high-volume hospitals. Numerous studies also assess this incipient centralization as a result of findings concerning the volume outcome relationship. For numerous types of interventions they describe a notable centralization process in US-American health care over a period of several years [20–25]. In other countries the findings on the volume outcome relationship have fed back into the development of medical care towards centralized and specialized care structures. The effects of such planned centralization strategies are the object of studies on health systems mainly in Europe and Japan [26–36]. These studies confirm without exception the positive correlation between high case volume and better treatment results. Nevertheless there is still some controversial debate on the pros and cons of centralization for specific interventions [37, 38]. Effects of prevalent centralization on patients are also a topic for research, with a focus on longer travel time for most and possible impairment of social support [22, 39–43].

Mandatory minimum volumes for certain defined surgical procedures were introduced in Germany in 2004 on a federal level. To date there are no studies into the implications of a possible centralization of care for these interventions. The minimum volume standards on the federal level that are mandatory for all approximately 2000 German acute-care hospitals apply to 6 types of surgical procedures, (1) complex esophageal interventions, (2) complex pancreatic interventions, (3) total knee replacement (TKR), and transplantations of (4) kidney, (5) liver and (6) stem cells. Between 2004 and 2006 these standards were raised in part and since then have remained the same (Table 1). Pertinent key figures for surgeries and procedures for each intervention are exactly defined in minimum volume regulations [44]. In Germany the Federal Joint Committee decides on minimum volume regulations. Represented in this body are the major actors of the health system, health insurance funds, physicians and hospitals; they form the joint self-government of the German health care system. Minimum volume regulations do not imply any scheduled structural revision of patient care with regard to the specific interventions, such as a systematic formation of centers and centralization. If hospitals perform minimum volume procedures they are obliged to register the case numbers in their quality reports as the only format to document compliance with minimum volume regulations.

**Table 1:** Specifications as per minimum volume regulations 2004 to 2012 for German hospitals: minimum volumes per hospital and year

Type of intervention (including)	2004	2006	2008	2010	2012
Complex esophageal interventions (partial/total esophagectomy, implant of magnetic antireflux device, gastrectomy with subtotal esophageal resection)	5	10	10	10	10
Complex pancreatic interventions (partial/total pancreatectomy, internal drainage of pancreas)	5	10	10	10	10
Kidney transplantation (re-transplantation)	20	25	25	25	25
Liver transplantation (re-transplantation, hepatectomy, partial hepatectomy from living donor)	10	20	20	20	20
Stem cell transplantation (transplantation of hematopoietic stem cells from bone marrow, transfusion of peripheral hematopoietic stem cells)	12	25	25	25	25
Total knee replacement	-	50	50	50	50

Quality reports are standardized nationwide, and all hospitals are legally required to publish their quality reports following the regulations on nature, scope and data format as defined by the Federal Joint Committee [45]. The quality report of each hospital in each reporting year consists of two data sets. One data set comprises structural and process data of the hospital, which are to be reported by the individual hospital following the regulations. These reported data are completely self-reported. A second data set for each hospital provides data on quality indicators as reported by each hospital to the official federal external quality assurance institution. These quality indicator data are validated in a structured manner including different statistical checks and a random sample of hospitals where data are verified during an on-site inspection each year. The data on minimum volume standards belong to the first data set without an external validation procedure. Hospital reports are publicly available on the Internet. An electronic data set of each reporting year can be obtained on application from the Federal Joint Committee. Reports on every second year of operation were mandatory from 2004 to 2012, and annual mandatory reports were introduced in 2013. Hospital quality reports for 2012 have been published in the summer of 2014.

In a preliminary evaluation of the consequences of the introduction of minimum volumes in German hospitals the authors reported earlier how many German hospitals performed the 5 procedures stipulated for 2004, how many complied with minimum volume standards, and indicated possible implications for geographically equal access to health care if all non-complying hospitals were excluded [46–49]. In 2014 the authors published a study updating the results on the research question how many hospitals complied with the minimum volume standards in each of the until then available hospital quality report data sets for the years 2004, 2006, 2008 and 2010 [50]. They could show that the number of hospitals not meeting the standard did not change over the years under study. The present study, however, does not focus on the number of hospitals not complying versus those complying with the standards but investigates the research question if from a health care system’s perspective a centralization occured over the time period from 2006 to 2010 with constant minimum volume standards (Table 1).

## Methods

The retrospective longitudinal analysis of secondary data is based on mandatory hospital quality reports for the years 2006, 2008 and 2010. Minimum volume data reported by hospitals are compared separately for the 6 types of interventions in the three years under review.

Centralization is operationalized for the purpose of this study as change over time in three respects: (1) the number of hospitals performing the respective procedure decreases over time; (2) the volume, i.e. number of cases, in high-volume hospitals and their percentage in the annual entire volume increase over time; (3) the volume in low-volume hospitals and their percentage in the annual entire volume decrease over time.

Hospital data on minimum volume procedures were exported from quality reports to EXCEL files for each year under review, whereby the number of performing hospitals and cases were descriptively registered for each minimum volume procedure and each year under review. For a comparison of annual volumes the hospitals were divided in quintiles (esophagus, pancreas, total knee replacement), and into tertiles for the three transplantation procedures. Hospitals were ranked according to number of performed procedures per type of intervention and year, and divided into exact quintile and tertile groups. A possible change in case distribution due to changing annual volumes and in the number of performing hospitals was taken into account in the formation of quintile and tertile groups by individual new rankings of groups for the years 2006, 2008 and 2010 respectively.

Volumes of quintile and tertile groups were analyzed statistically for significant changes within each quintile and tertile over the three years under review (intra quartile / intra tertile differences). Subsequently the relative percentage ratios between quintiles / tertiles of one year were analyzed over the three one-year periods (inter quartile / inter tertile differences). The intra quartile / intra tertile volume change over the three years was submitted to test-statistical analysis using the Kruskal Wallis test for independent, non-parametric testing of samples, since the ranked quintile and tertile groups were assumed to be not normally distributed. Test-statistical analysis of inter quartile/inter tertile differences in case numbers over the three years under review was checked with a chi-square test for each minimum volume. The statistics software IBM SPSS Statistics Version 21 was used for test-statistical analysis.

### Ethics statement

The authors declare that their study does not need ethical approval. All data used in this study are publicly available and usable due to legal obligations of hospitals to provide a high standard of transparency and offer information for patients, hospitals, health insurers, research and politics. Single hospital quality reports can be accessed via: http://www.g-ba-qualitaetsberichte.de/. Electronic data sets (XML format) of the quality reports can be requested from the Federal Joint Committee via: https://www.g-ba.de/institution/themenschwerpunkte/qualitaetssicherung/qualitaetsbericht/xml-daten/.

## Results

### Number of hospitals per minimum volume and compliance with minimum volume standards over time

The basic data on the number of hospitals and cases per year and minimum volume are listed in Tables 2 to 7 in the upper part respectively. From 2006 to 2010, the number of hospitals performing pancreatic, esophageal and total knee replacement interventions increased by 4 to 11 %, and the annual volume increased by 8 to 25 %. For all three minimum volumes the number of hospitals complying with the minimum volume standards remained constant or increased slightly. A major difference is notable in the percentage of complying hospitals between pancreatic interventions (70 %), esophageal interventions (55 %) and total knee replacement (90 %). For the volume, the percentage of cases in hospitals meeting minimum volume standards is between 85 and 99 %. The number of hospitals for stem cell and liver transplantations is constant over time, whereas a slight decrease has been registered for kidney transplantations. The percentage of hospitals complying with minimum volume standards is over 90 % for kidney and liver transplantations, but for stem cell transplantations is 75 % with a downward trend. For the cases treated in these hospitals the percentage is over 95 % respectively.

**Table 2:** Number of hospitals and cases for complex pancreatic interventions in 2006, 2008, and 2010 and number of cases per quintile

	2006	2008	2010	p
Hospitals with no. of cases ≥ 1	437	444	461	
Change to 2006	-	+1,6 %	+5,5 %	
Annual no. of cases	8258	8843	9180	
Change to 2006	-	+7,1 %	+11,2 %	
Min. – max.	1–384	1–395	1–427	
Median	11,00	13,00	13,00	
Mean (SD)	18,90 (31,6)	19,92 (29,3)	19,91 (29,6)	
Hospitals complying MVS*: 10 per year	279 (63,8 %)	311 (70,0 %)	328 (71,1 %)	
No. of cases in hospitals complying MVS	7534 (91,2 %)	8236 (93,1 %)	8576 (93,4 %)	
Hospitals not complying MVS	158 (36,2 %)	133 (30,0 %)	133 (28,9 %)	
No. of cases in hospitals not complying MVS	724 (8,8 %)	607 (6,9 %)	604 (6,6 %)	
Quintile 1 hospitals	87	88	92	
No. of cases	4794	4938	5087	0,735**
Percentage of annual cases	58,0 %	55,8 %	55,4 %	
Min. – max.	25–384	25–395	27–427	
Median	38,00	40,00	39,00	
Mean (SD)	55,10 (57,0)	56,11 (50,6)	55,29 (51,8)	
Quintile 2 hospitals	87	89	92	
No. of cases	1550	1682	1770	0,002**
Percentage of annual cases	18,8 %	19,0 %	19,3 %	
Min. – max.	14–25	15–25	15–26	
Median	17,00	18,00	18,00	
Mean (SD)	17,82 (3,4)	18,90 (2,5)	19,24 (3,2)	
Quintile 3 hospitals	87	89	92	
No. of cases	1010	1152	1182	<0,001**
Percentage of annual cases	12,2 %	13,0 %	12,9 %	
Min. – max.	10–14	11–15	11–15	
Median	12,00	13,00	13,00	
Mean (SD)	11,61 (1,1)	12,94 (1,3)	12,85 (1,3)	
Quintile 4 hospitals	88	89	92	
No. of cases	673	802	860	<0,001**
Percentage of annual cases	8,1 %	9,1 %	9,4 %	
Min. – max.	5–10	6–11	7–11	
Median	8,00	10,00	10,00	
Mean (SD)	7,65 (1,6)	9,01 (1,5)	9,35 (1,3)	
Quintile 5 hospitals	88	89	93	
No. of cases	231	269	281	0,295**
Percentage of annual cases	2,8 %	3,0 %	3,1 %	0,999#
Min. – max.	1–5	1–6	1–6	
Median	3,00	3,00	3,00	
Mean (SD)	2,63 (1,4)	3,02 (1,8)	3,02 (1,7)	

*minimum volume standards; **Kruskal-Wallis-test; # chi-square-test

### Caseload change over time

Tables 2, 3, 4, 5, 6 and 7 present results for caseload changes in each quintile/tertile over the three years under review. For pancreatic interventions (Table 2), the caseloads increased significantly over time from the 2nd quintile (hospital group with the second most frequent volumes) up to the 4th quintile (hospital group with second-lowest volume); in the 1st quintile (hospital group with most frequent volume) the percentage of cases declined slightly and in the 5th quintile (hospital group with lowest volumes) it slightly rose. For esophageal interventions (Table 3), the caseload in each quintile did not change significantly, except for the 3rd quintile (hospital group with medium volumes) where it declined with *p* = 0,040. Total knee replacement interventions (Table 4) showed a significant increase in volume over all quintiles. For all three transplantation procedures (Tables 5, 6 and 7) the volume remained constant in tertile groups over time. Statistical analysis of the relative percentage of case distribution between the three years under review over all quintiles / tertiles of each minimum volume reveals p values of over 0.99 % in the chi-square test for all minimum volumes. This corresponds virtually to a constant relative volume distribution for all minimum volumes over the period under consideration.

**Table 3:** Number of hospitals and cases for complex esophageal interventions in 2006, 2008, and 2010 and number of cases per quintile

	2006	2008	2010	p
Hospitals with no. of cases ≥ 1	271	275	283	
Change to 2006	-	+1,5 %	+4,4 %	
Annual no. of cases	3315	3422	3576	
Change to 2006	-	+3,2 %	+7,9 %	
Min. – max.	1–150	1–124	1–112	
Median	10,00	10,00	10,00	
Mean (SD)	12,23 (13,8)	12,44 (14,1)	12,64 (14,2)	
Hospitals complying MVS*: 10 per year	154 (56,8 %)	148 (53,8 %)	160 (56,5 %)	
No. of cases in hospitals complying MVS	2809 (84,7 %)	2875 (84,0 %)	3066 (85,7 %)	
Hospitals not complying MVS	117 (43,2 %)	127 (46,2 %)	123 (43,5 %)	
No. of cases in hospitals not complying MVS	506 (15,3 %)	547 (16,0 %)	510 (14,3 %)	
Quintile 1 hospitals	54	55	57	
No. of cases	1636	1800	1857	0,302**
Percentage of annual cases	49,4 %	52,6 %	51,9 %	
Min. – max.	16–150	17–124	17–112	
Median	22,50	24,00	23,00	
Mean (SD)	30,30 (22,0)	32,73 (20,3)	32,58 (20,7)	
Quintile 2 hospitals	54	55	56	
No. of cases	696	687	729	0,165**
Percentage of annual cases	21,0 %	20,1 %	20,4 %	
Min. – max.	11–16	11–16	11–17	
Median	12,50	12,00	12,00	
Mean (SD)	12,89 (1,5)	12,49 (1,6)	13,02 (1,8)	
Quintile 3 hospitals	55	55	57	
No. of cases	557	537	569	0,040**
Percentage of annual cases	16,8 %	15,7 %	15,9 %	
Min. – max.	8–11	8–11	8–11	
Median	10,00	10,00	10,00	
Mean (SD)	10,13 (0,7)	9,76 (0,8)	9,98 (0,6)	
Quintile 4 hospitals	54	55	56	
No. of cases	321	297	325	0,092**
Percentage of annual cases	9,7 %	8,7 %	9,1 %	
Min. – max.	4–8	4–8	3–8	
Median	6,00	5,00	6,00	
Mean (SD)	5,94 (1,3)	5,40 (1,3)	5,80 (1,4)	
Quintile 5 hospitals	54	55	57	
No. of cases	105	101	96	0,405**
Percentage of annual cases	3,2 %	3,0 %	2,7 %	0,999#
Min. – max.	1–4	1–4	1–3	
Median	2,00	2,00	2,00	
Mean (SD)	1,94 (0,9)	1,84 (1,0)	1,68 (0,8)	

*minimum volume standards; **Kruskal-Wallis-test; # chi-square-test

**Table 4:** Number of hospitals and cases for total knee replacement interventions in 2006, 2008, and 2010 and number of cases per quintile

	2006	2008	2010	p
Hospitals with no. of cases ≥ 1	855	905	949	
Change to 2006	-	+5,8 %	+11,0	
Annual no. of cases	114852	139287	143593	
Change to 2006	-	+21,3 %	+25 %	
Min. – max.	1–979	1–1329	1–1367	
Median	93,00	109,00	107,00	
Mean (SD)	134,33 (126,7)	153,91 (144,1)	151,31 (140,4)	
Hospitals complying MVS*: 50 per year	745 (87,1 %)	834 (92,2 %)	870 (91,7 %)	
No. of cases in hospitals complying MVS	112305 (97,8 %)	137586 (98,8 %)	141597 (98,6 %)	
Hospitals not complying MVS	110 (12,9 %)	71 (7,8 %)	79 (8,3 %)	
No. of cases in hospitals not complying MVS	2547 (2,2 %)	1701 (1,2 %)	1996 (1,4 %)	
Quintile 1 hospitals	171	181	190	
No. of cases	56617	67795	69609	0,001**
Percentage of annual cases	49,3 %	48,7 %	48,5 %	
Min. – max.	191–979	215–1329	219–1367	
Median	276,00	318,00	309,50	
Mean (SD)	331,09 (153,4)	374,56 (181,9)	366,36 (177,0)	
Quintile 2 hospitals	171	181	190	
No. of cases	25650	30686	31322	<0,001**
Percentage of annual cases	22,3 %	22,0 %	21,8 %	
Min. – max.	117–190	133–215	130–218	
Median	147,00	168,00	163,50	
Mean (SD)	150,00 (21,3)	169,54 (24,3)	164,85 (23,7)	
Quintile 3 hospitals	171	181	189	
No. of cases	16051	19870	20635	<0,001**
Percentage of annual cases	14,0 %	14,3 %	14,4 %	
Min. – max.	75–116	89–153	91–130	
Median	93,00	109,00	107,00	
Mean (SD)	93,87 (11,9)	109,78 11,5)	109,18 (11,7)	
Quintile 4 hospitals	171	181	190	
No. of cases	10857	13302	14042	<0,001**
Percentage of annual cases	9,5 %	9,6 %	9,8 %	
Min. – max.	53–75	60–89	59–91	
Median	63,00	74,00	73,00	
Mean (SD)	63,49 (6,7)	73,49 (8,1)	73,91 (9,2)	
Quintile 5 hospitals	171	181	190	
No. of cases	5677	7634	7985	<0,001**
Percentage of annual cases	4,9 %	5,5 %	5,6 %	0,999#
Min. – max.	1–53	1–60	1–59	
Median	40,00	51,00	51,00	
Mean (SD)	33,20 (18,1)	42,18 (18,0)	42,03 (17,6)	

*minimum volume standards; **Kruskal-Wallis-test; # chi-square-test

**Table 5:** Number of hospitals and cases for kidney transplantations in 2006, 2008, and 2010 and number of cases per tercile

	2006	2008	2010	p
Hospitals with no. of cases ≥ 1	40	40	37	
Change to 2006	-	0,0 %	−7,5 %	
Annual no. of cases	2784	2784	2856	
Change to 2006	-	0,0 %	+2,6 %	
Min. – max.	4–251	9–255	12–264	
Median	57,50	60,50	69,00	
Mean (SD)	69,60 (48,4)	69,60 (48,7)	77,19 (51,7)	
Hospitals complying MVS*: 25 per year	38 (95 %)	36 (90,0 %)	35 (94,6 %)	
No. of cases in hospitals complying MVS	2769 (99,5 %)	2721 (97,7 %)	2824 (98,9 %)	
Hospitals not complying MVS	2 (5,0 %)	4 (10,0 %)	2 (5,4 %)	
No. of cases in hospitals not complying MVS	15 (0,5 %)	63 (2,3 %)	32 (1,1 %)	
Tercile 1 hospitals	13	13	12	
No. of cases	1581	1597	1598	0,438**
Percentage of annual cases	56,8 %	57,3 %	56,0 %	
Min. – max.	85–251	86–255	96–264	
Median	105,00	107,00	117,00	
Mean (SD)	121,62 (48,3)	122,85 (47,5)	133,17 (51,0)	
Tercile 2 hospitals	14	14	13	
No. of cases	837	830	883	0,288**
Percentage of annual cases	30,1 %	29,8 %	30,9 %	
Min. – max.	39–84	39–84	40–94	
Median	57,50	60,50	69,00	
Mean (SD)	59,79 (16,1)	59,29 (15,1)	67,92 (15,6)	
Tercile 3 hospitals	13	13	12	
No. of cases	366	357	375	0,433**
Percentage of annual cases	13,1 %	12,8 %	13,1 %	0,999#
Min. – max.	4–38	9–38	12–39	
Median	32,00	30,00	33,50	
Mean (SD)	28,15 (10,4)	27,46 (9,0)	31,25 (8,0)	

*minimum volume standards; **Kruskal-Wallis-test; # chi-square-test

**Table 6:** Number of hospitals and cases for stem cell transplantations in 2006, 2008, and 2010 and number of cases per tercile

	2006	2008	2010	
Hospitals with no. of cases ≥ 1	81	73	84	
Change to 2006	-	−9,9 %	+2,5 %	
Annual no. of cases	6181	5542	6320	
Change to 2006	-	−10,3 %	+2,2 %	
Min. – max.	1–349	1–306	1–320	
Median	40,00	40,00	38,50	
Mean (SD)	76,31 (78,9)	75,92 (74,9)	75,24 (77,5)	
Hospitals complying MVS*: 25 per year	63 (77,8 %)	55 (75,3 %)	62 (73,8 %)	
No. of cases in hospitals complying MVS	5927 (95,9 %)	5290 (95,5 %)	6030 (95,4 %)	
Hospitals not complying MVS	18 (22,2 %)	18 (24,7 %)	21 (26,2 %)	
No. of cases in hospitals not complying MVS	254 (4,1 %)	252 (4,5 %)	290 (5,6 %)	
Tercile 1 hospitals	27	24	28	
No. of cases	4497	3940	4694	0,904**
Percentage of annual cases	72,8 %	71,1 %	74,3 %	
Min. – max.	80–349	83–306	82–320	
Median	143,00	153,00	158,00	
Mean (SD)	166,56 (76,1)	164,17 (67,1)	167,64 (167,6)	
Tercile 2 hospitals	27	24	28	
No. of cases	1202	1170	1184	0,381**
Percentage of annual cases	19,4 %	21,1 %	18,7 %	
Min. – max.	27–79	27–82	26–82	
Median	40,00	40,50	38,50	
Mean (SD)	44,52 (16,0)	48,75 (19,3)	42,29 (16,1)	
Tercile 3 hospitals	27	25	28	
No. of cases	482	432	442	0,547**
Percentage of annual cases	7,8 %	7,8 %	7,0 %	0,990#
Min. – max.	1–26	1–26	1–26	
Median	19,00	19,00	16,00	
Mean (SD)	17,85 (7,6)	17,28 (8,5)	15,79 (7,7)	

*minimum volume standards; **Kruskal-Wallis-test; # chi-square-test

**Table 7:** Number of hospitals and cases for liver transplantations in 2006, 2008, and 2010 and number of cases per tercile

	2006	2008	2010	
Hospitals with no. of cases ≥ 1	23	23	23	
Change to 2006	-	0,0 %	0,0 %	
Annual no. of cases	1381	1386	1464	
Change to 2006	-	+0,4 %	+6,0 %	
Min. – max.	16–143	11–146	17–160	
Median	51,00	47,00	60,00	
Mean (SD)	60,04 (36,7)	60,26 (37,9)	63,65 (35,7)	
Hospitals complying MVS*: 20 per year	22 (95,7 %)	20 (87,0 %)	22 (95,7 %)	
No. of cases in hospitals complying MVS	1365 (98,8 %)	1349 (97,3 %)	1447 (98,8 %)	
Hospitals not complying MVS	1 (4,3 %)	3 (13,0 %)	1 (4,3 %)	
No. of cases in hospitals not complying MVS	16 (1,2 %)	37 (2,7 %)	17 (1,2 %)	
Tercile 1 hospitals	7	7	7	
No. of cases	739	748	749	0,989**
Percentage of annual cases	53,5 %	54,0 %	51,2 %	
Min. – max.	65–143	80–146	90–160	
Median	103,00	102,00	97,00	
Mean (SD)	105,57 (29,6)	106,86 (24,4)	107,00 (24,4)	
Tercile 2 hospitals	8	8	8	
No. of cases	426	444	483	0,428**
Percentage of annual cases	30,8 %	32,0 %	33,0 %	
Min. – max.	46–60	42–70	43–81	
Median	53,50	55,50	60,00	
Mean (SD)	53,25 (5,4)	55,50 (13,3)	60,38 (12,2)	
Tercile 3 hospitals	8	8	8	
No. of cases	216	194	232	0,578**
Percentage of annual cases	15,6 %	14,0 %	15,8 %	0,990#
Min. – max.	16–41	11–41	17–43	
Median	24,50	20,50	28,50	
Mean (SD)	27,00 (8,5)	24,25 (12,5)	29,00 (9,4)	

*minimum volume standards; **Kruskal-Wallis-test; # chi-square-test

## Discussion

No centralization of care was found in the German hospital sector for the 6 minimum volume interventions from 2006 to 2010. The three criteria for centralization - less hospitals perform an intervention, growing case load and higher percentage of cases in high-volume hospitals, and declining case load and lower percentage of cases in low-volume hospitals – do not apply to any of the six types of interventions. On the contrary, the percentage of hospitals performing the respective minimum volume procedures rose for four of the procedures by between 2.5 and 11 % over the 5-year period, remained constant only for liver transplantations, and declined for kidney transplantations. The percentage of the annual distribution of volume percentages between the quintiles/tertiles is virtually identical for all minimum volumes over the three years under review. The increase in caseload for all 6 procedures did not affect centralization tendencies. The strong increase in case loads for pancreatic (11 %), esophageal (8 %) and total knee replacement (25 %) interventions over the 5-year period resulted in a significant increase over all quintiles for total knee replacement, and over several of the intermediate quintiles for the two other procedures. For these three procedures the volume increase is higher than the average 7.3 % increase for all in-patient admissions to German hospitals from 2006 to 2010.

What are possible reasons why no centralization occured in any of the introduced minimum volume interventions? We assume different contributing factors in each of the procedures. Liver and kidney transplantation, with 23 and 40 delivering centers nationwide respectively, were already highly centralized before introducing minimum volume standards and no further centralization was expected [46]. Stem cell transplantations are a special case in this context, since they are not subject to regulations for organ transplants as liver and kidney transplantation. Their indication is governed by dynamic developments in medicine, as may be seen from volatile figures for hospitals and case loads over the three years under study, with a tendency towards more hospitals, and cases performed without meeting minimum standards. The high reimbursements may be an additional incentive for performing such interventions. Total knee replacements are very frequent interventions, performed in every second German hospital, and have increased by 25 % within 5 years. The currently applicable minimum volume of 50 procedures per year must be considered as low to medium, based on the systematic review by Marlow [13]. The caseloads in German hospitals not meeting minimum volume standards are – with 1 to 2 % - as low as only those for centralized liver and kidney transplantations.

In contrast, pancreatic and esophageal interventions had the highest percentage of hospitals not complying with minimum volume standards in 2006 with 36 and 43 % respectively, and a centralization was expected [46, 51, 52]. Our findings on these two procedures can be contrasted by international studies quoted earlier. Numerous US studies explore the introduction of minimum volumes and the centralization processes involved for these two interventions among others. Based on a nationwide inpatient sample, Learn [20] reports that 67 % of pancreatectomies were performed in hospitals with less than 10 such interventions per year from 1997 to 1999; the percentage declined to 51 % from 2004 to 2006, comparable to esophagectomies with an initial percentage of 69 % in hospitals with less than 6 cases per year where the percentage declined to 53 %. For these two intervention categories Stitzenberg [39] also reports a shift of cases towards high-volume hospitals, based on data collected in three US states. Between 1996 and 2006 the percentage of esophagectomies performed by hospitals with 8 or less procedures per year declined from 56 to 23 %, and the percentage of pancreatectomies from 63 to 31 % in hospitals with 14 or less procedures per year. For Washington State, Massarweh [24] describes a decline in pancreatectomies from 41 % in 1994 to 24 % in 2007 in hospitals with 10 or fewer procedures per year, and from 58 to 41 % for esophagectomies in hospitals with 12 or fewer procedures per year. He explicitly refers to the minimum volume requirements postulated by the Leapfrog Group as a driving force. Two aspects about these data trends in the US are noteworthy in comparison to Germany. Over the (longer) periods under review of 9 to 13 years a centralization of these interventions is observable in the United States. The achieved shifts towards high-volume hospitals are, however, still nowhere near from meeting the goal of complete or even high-level centralization. In Germany the two procedures in question were already more centralized upon introduction of minimum volume standards, and in 2010 only 14 % of esophagectomies and 7 % of pancreatectomies were performed by hospitals with less than 10 such surgeries per year.

From the Netherlands de Wilde [31] and Wouters [32] report the successful implementation of a minimum volume of 10 pancreatectomies per year, with a decrease in performing hospitals from 48 to 30, and an increase from 53 to 91 % in patients operated upon in hospitals with 10 and more interventions. Centralization criteria have been clearly met in this case. From the south of the Netherlands Gooiker [33] and Lemmens [34] give an impressive description of an inter-hospital, coordinated centralization of esophagectomies and pancreatectomies, leading to performance in 2 instead of 9 hospitals and to double-digit volumes.

These international experiences with introducing minimum volume standards for esophagectomies and pancreatectomies can help understanding the differing development in Germany. The low percentage of esophagectomies and pancreatectomies in German hospitals with less than 10 interventions per year reflects - in international comparison - a high degree of centralization existing prior to the introduction of minimum volume standards. Linked to this is a not equally promoted compliance with minimum volume standards on the part of medical societies, national specialist institutes or initiatives such as Leapfrog with an influence on reimbursement criteria in other countries. German minimum volume regulations oblige hospitals to comply but do not specify any sanctions. A 2006 survey among affected German hospitals not complying with minimum volumes for esophagectomies and pancreatectomies, and also among national umbrella organizations of all German sickness funds revealed that non-compliance has so far been irrelevant in the annual budget negotiations [53]. Another factor with impact on centralization processes in Germany is its federal structure. The 16 federal states in Germany are independently in charge of designing and implementing the structural planning of inpatient care, and not the Federal Joint Committee that defines nationwide minimum volume standards. This might be one important factor why on the state level in North-Rhine Westphalia breast cancer treatment was successfully concentrated in breast cancer centers, thereby reducing the number of hospitals with breast cancer treatment from about 250 to 100 [54]. This approach is comparable to The Netherlands where relocation of procedures as basic step toward centralization has been successfully fostered by specifying care structures and by cooperation in regional quality associations [31–33].

However, factors such as promoting initiatives of medical societies, reimbursement strategies, sanctions, administrative directives, and regional quality associations focus primarily on a system perspective. It might be helpful to address as well the hospital perspective to achieve stricter compliance with minimum volume standards. Especially hospitals not complying with minimum volume standards should be analysed why such procedures are performed. Factors to be studied are the relevance of and options for emergency procedures, attitudes towards and possibilities of regional cooperation and last but not least the patient view, since proximity to the place of residence and therefore to social support have a high priority for which patients are willing to accept higher risks [55].

### Limitations

A limitation of this study is that the validity of data used from hospital quality reports is not quantifiable. The data are unevaluated and self-reported. It has been established that the reported data have had no practical consequences in the form of sanctions to date. But the possibility cannot be ruled out that data reported specifically from hospitals not meeting minimum volumes are inaccurate. The study does not investigate the temporal, intrahospital volume progression that would be of interest for individual hospital developments in complementing the systemic perspective of centralization, nor does it consider the influence of cooperation between hospitals on volume progression in detail. From the perspective of the individual hospital, the emergence of other regional health care structures may constitute important developments after the introduction of minimum volume standards, but has not been explored for the purposes of this study.

## Conclusions

The nationwide introduction of minimum volume standards in German hospitals for 6 procedures did not result in centralization. If the aim is to achieve better compliance with minimum volume standards, reasons for non-compliance from the hospital and the patient perspective must be analyzed in greater detail and addressed accordingly.
